# Evaluation of Rare Hand Injection Injuries With Three Different Substances

**DOI:** 10.1155/cris/9941563

**Published:** 2026-04-28

**Authors:** Hasan Basri Çağlı, Salih Can Sezer, Abdurrahman Ercan Yurttaşer, Ahmet Özdemir, Tahir Babahan, Merve Özger, Mustafa Yılmaz

**Affiliations:** ^1^ Department of Plastic Reconstructive and Aesthetic Surgery, Dokuz Eylul University, Izmir, Türkiye, deu.edu.tr

**Keywords:** grease injection, hand surgery, injection injury, mercury injection, paint injection

## Abstract

Injection injuries to the hand are rare and often underestimated in the initial evaluation due to minor external findings and mild symptoms. However, these injuries can lead to local and systemic complications with mechanical and toxic damage mechanisms. This study presents three cases of hand injection injuries caused by different substances.

## 1. Introduction

Injection injuries to the hand are rare and can result in varying degrees of loss of function, ranging from permanent sensory deficits to amputation. Previous studies report amputation rates ranging approximately from 16% to 48%, depending on factors such as the injected material, injection pressure, injury location, and timing of surgical treatment [1–3]. The mechanism of damage can be described as mechanical and toxic damage. In addition to these, secondary infections may become problematic, leading to tissue destruction and damage spread [4, 5].

Mechanical damage may be related to injection pressure and the mass effect of the injected material between tissue compartments. Hart et al. [3] reported that significant mechanical damage occurred when the injection pressure was between 2000 and 12,000 psi; Hogan et al.[2] reported amputation rates as high as 43% for pressure injuries above 1000 psi and 19% for injuries below 1000 psi. The mass effect varies depending on the amount injected, the injection site, and the distribution area of the substance. Injected material typically spreads along the path of least resistance after a small puncture in the skin [6, 7]. Increasing the injection volume, finger injections, and spreading into the neurovascular area will subsequently increase the damage.

Toxic damage is often related to the extent of the local inflammatory response. This inflammatory response can cause compression of local neurovascular structures, resulting in vasospasm, thrombosis, ischemia and necrosis [3]. Injuries from organic sources such as paint thinner, paint, diesel fuel, gasoline, jet fuel and petroleum are the most toxic; hydraulic fluid and grease are less toxic; and water and air injection are nontoxic but create mechanical damage [2]. In addition to local toxicity, some substances cause severe systemic toxicity. Injection injuries from substances with high systemic toxicity can occur accidentally, as suicide attempts, as part of drug addiction and in cases of psychiatric illness. These substances can cause central nervous system, pulmonary, cardiac, gastrointestinal, and renal toxicity. Hand injection of substances such as mercury and hydrocarbons, which cause high systemic toxicity, has been reported in the literature [8–14].

In this study, we aimed to evaluate three injection injuries to the hand with three different substances and to contribute to the literature on managing injection injuries to the hand.

## 2. Case 1

**Figure 1:**
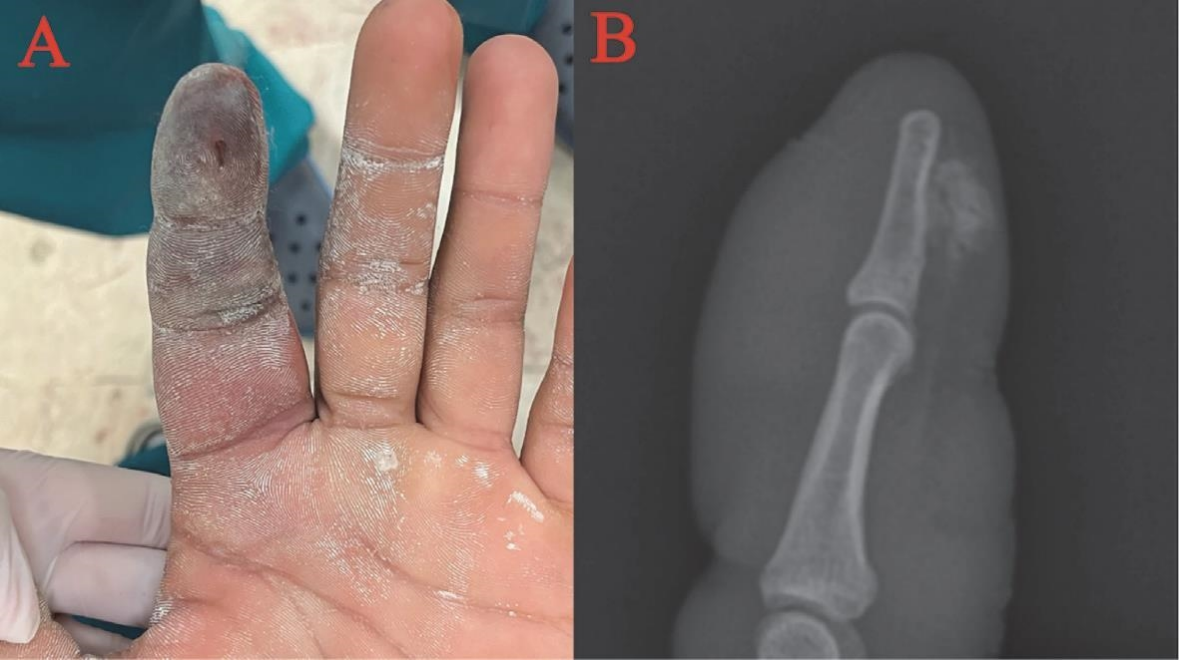
Photographs of Case 1. (A) Circulatory impairment of the finger on the third day after paint‐gun injection injury. (B) Radiopaque material visible on X‐ray.

A 22‐year‐old right‐handed male patient presented 3 days after the injury, following a paint‐gun injection injury to the second finger, with edema, pain, and poor circulation in the finger (Figure 1A). The patient had a small puncture injury to the pulp, signs of local infection extending to the metacarpophalangeal joint, and hemorrhagic bullae and partial necrosis in the distal phalanx. Laboratory findings were unremarkable except for elevated CRP and WBC. X‐ray showed radiopaque material in the distal phalanx (Figure 1B).

The patient was admitted and started on broad‐spectrum antibiotics. Incision and drainage (I&D) were performed on the day of admission (third day postinjury), and catheter irrigation was also used. The infection cleared 3 days after treatment, and the patient was discharged. After necrosis demarcation was completed 3 weeks later, the foreign material was fully removed, and distal phalanx amputation was performed.

## 3. Case 2

**Figure 2:**
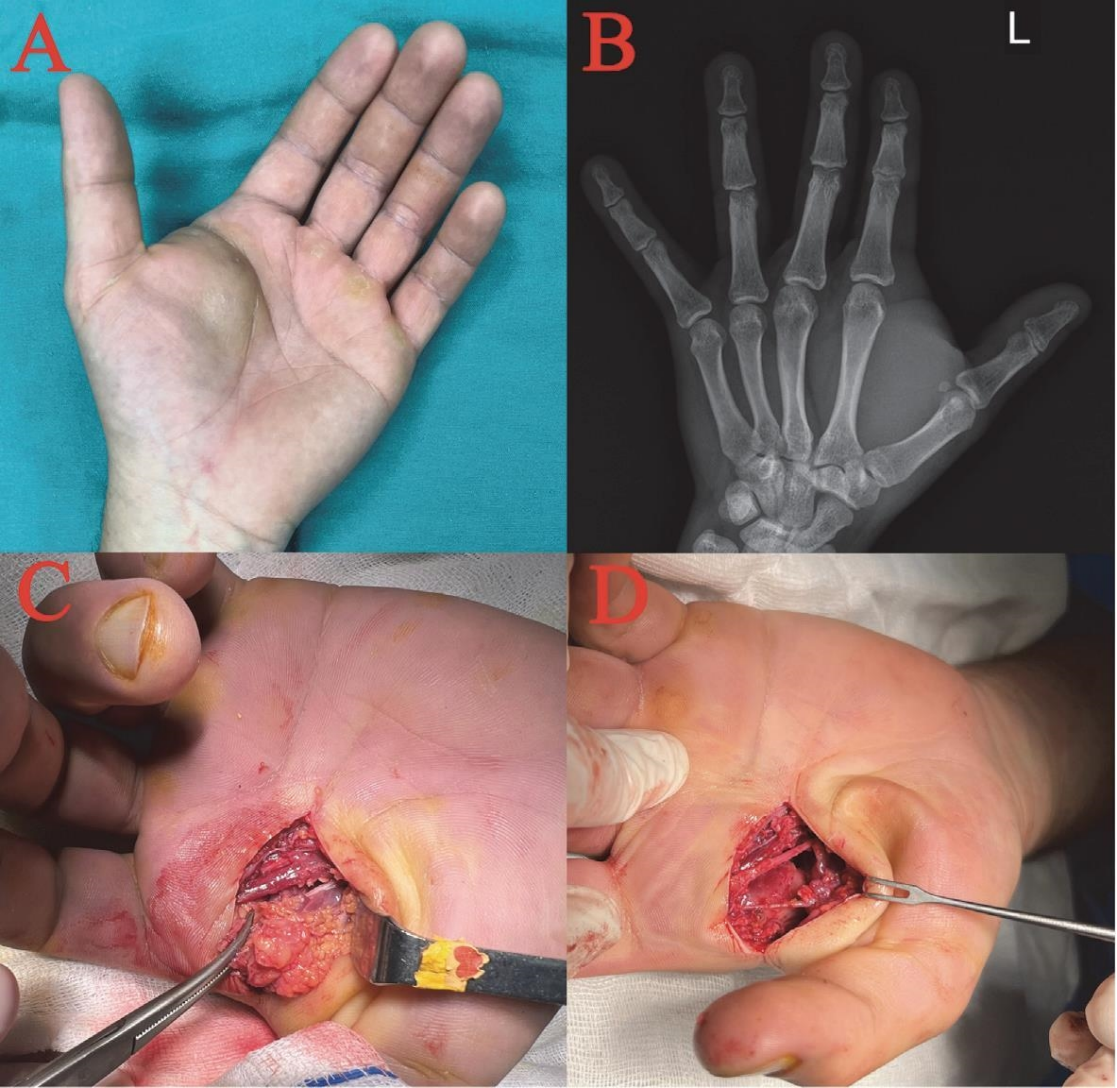
Photographs of Case 2. (A) Mass formation in the thenar region on the 15th day after a grease injection injury. (B) No radiopaque material visible on X‐ray. (C) Intraoperative view showing necrotic tissue and muscle degeneration. (D) Intraoperative view after debridement with preserved neurovascular structure.

A 30‐year‐old right‐handed male patient was admitted to a medical center after a grease injection injury to the thenar region of the left hand. Initially, the severity of the injury was underestimated due to a small puncture injury, no X‐ray findings and mild symptoms. Consequently, he was followed as an outpatient. On the 15th day of the injury, the patient presented to our hospital with swelling and persistent pain extending dorsally from the web space in the thenar region (Figure 2A). There were no additional findings on examination other than induration in the swollen area and pain‐related limited range of motion. X‐ray findings showed no radiopaque material (Figure 2B). Additional imaging studies were then performed. Ultrasonography (US) revealed hyperechogenic, mass‐like areas in the thenar region. Contrast‐enhanced magnetic resonance imaging (MRI) showed foreign material deposits and signal‐free areas within muscles, consistent with necrosis.

Broad‐spectrum antibiotic therapy was started after the patient’s admission, and the patient was taken to debridement surgery under general anesthesia on the same day (15 days postinjury). Extensive debridement was performed with two separate incisions in the thenar and dorsal regions. During surgery, foreign material was seen between the tissue planes, and thenar muscles showed degenerative areas in addition to a fibrotic mass (Figure 2C). The radial neurovascular structure of the 2nd digit was dissected and preserved (Figure 2D). A catheter was placed, and daily irrigation was performed for 5 days. On day 3, the culture was negative, and antibiotic therapy was discontinued. The patient was discharged after 7 days of hospitalization. Pathologic evaluation showed foreign body reaction and areas of fat necrosis.

## 4. Case 3

**Figure 3:**
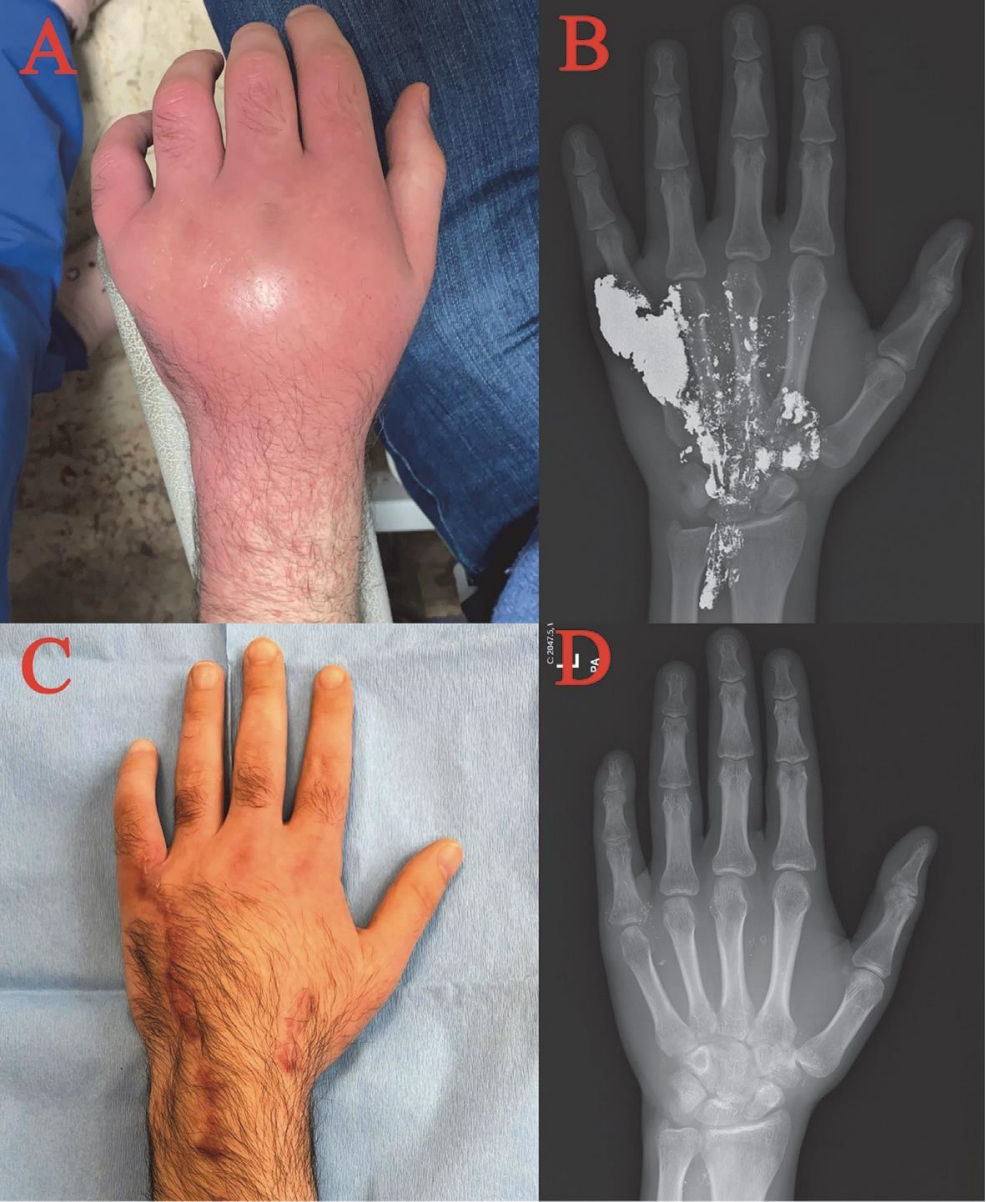
Photographs of Case 3. (A) Edematous and erythematous appearance of the hand on the seventh day after subcutaneous injection of elemental mercury. (B) Radiopaque material visible on X‐ray. (C) Clinical appearance at the fourth postoperative month. (D) Postoperative X‐ray.

A 31‐year‐old right‐handed male patient with known diagnoses of attention deficit hyperactivity disorder and multiple sclerosis presented with complaints of pain, swelling, edema, and erythema of the hand, reportedly following an insect bite on the left hand 1 week ago (Figure 3A). The patient’s evaluation revealed high fever, localized infection, and motor and sensory deficits in the left hand and forearm, as well as diffuse radiopaque areas on X‐ray (Figure 3B). Upon elaboration of the anamnesis, the patient stated that he used elemental mercury in his house. The patient’s blood mercury level was 647 μg/L (normal range 0.46–7.5), and 24‐h urine mercury level was 5514 μg.

2,3‐Dimercaptosuccinic acid (DMSA) was started as chelation therapy, and clindamycin and ciprofloxacin were started as antibiotic therapy. Surgical treatment was performed on the admission day (1 week postinjury) to reduce the mercury load and control the local tissue damage with biosecurity measures. Mercury particles were observed in multiple tissue planes, suggesting that mercury may have been injected more than once. Extensive debridement was performed with tendon preservation. Intraoperative fluoroscopy confirmed near‐complete removal of mercury‐related radiopaque material.

During follow‐up, the patient developed respiratory distress during follow‐up and was treated for mercury embolism. The patient’s systemic symptoms and pulmonary infiltration findings resolved, and good wound healing of the hand was observed with respiratory support therapy, chelation, antibiotic therapy, and surgical treatment (Figure 3C, D).

The patient was hospitalized for 17 days under a multidisciplinary team, including plastic surgery, infectious diseases, pharmacology, hematology, neurology, thoracic diseases, nephrology, psychiatry, dermatology, and physical therapy and rehabilitation. At follow‐up, the patient had no significant or only partial functional limitation. Blood mercury levels at 2 and 3 months were 39.3 μg/L and 27 μg/L (normal range 0.46–7.5), respectively, and 24‐h urine mercury levels were 51.7 μg and 59.2 μg, respectively.

The key clinical features, imaging findings, treatments, and outcomes of the three cases are summarized in Table 1.

**Table 1:** Clinical characteristics, management, and outcomes of the three hand injection injury cases.

Case	Substance	Injury site	Time to presentation (days)	Imaging findings	Surgical treatment	Complications	Outcome
1	Paint	Second finger pulp	3	Radiopaque material on X‐ray	Incision and drainage + catheter irrigation + distal phalanx amputation	Local necrosis	Good healing
**2**	Grease	Thenar region	15	MRI and US showing foreign material	Extensive debridement + catheter irrigation	Muscle degeneration	Functional recovery
3	Mercury	Hand	7	Radiopaque material on X‐ray	Debridement + chelation	Mercury embolism	Improved motor function

## 5. Discussion

Injection injuries to the hand are often underestimated during the initial evaluation because of minimal external findings and mild symptoms. However, mechanical and toxic damage, in addition to secondary infections, may lead to systemic pathologies and loss of limb. The location, injection pressure, volume, and local and systemic toxicity of the material should be carefully evaluated to determine the extent of injury and guide management [1–4, 6].

Paint gun injection is one of the most common and most toxic injuries. Paint consists of three primary components: binder, pigment, and solvent [2, 5, 11]. Oil‐based paints are more toxic than latex‐based paints and carry a higher risk of amputation [2]. X‐rays are almost always useful. The general approach is debridement within the first 6 h [2, 4, 7]. However, in patients presenting late, a more conservative initial approach aimed at infection control may be considered. Extensive traumatic surgery involving the area of infection may increase ischemia and the area of necrosis [15]. In the first case, X‐ray findings showed a paint substance limited to the distal phalanx with necrosis and the proximal infection was controlled with antibiotic therapy, I&D and catheter irrigation. In the follow‐up, amputation of the distal phalanx with necrosis demarcation was performed, and the paint was completely removed with amputation.

Grease gun injection is also one of the most common injection injuries. It causes relatively less local toxicity than paint. Depending on its content, grease oil may be radiopaque or radiolucent on X‐ray. If foreign material injection is suspected but no foreign material is seen on X‐ray, MRI, and US may be useful [16]. In the second case we present, the patient’s injury was underestimated due to a small puncture wound, mild symptoms and absence of X‐ray findings. However, further investigation with US and MRI showed a foreign material injury. Although grease injections are generally associated with lower toxicity, early surgical exploration is still recommended in most cases to prevent compartment pressure increase, foreign material spread, and chronic inflammatory reactions [2, 3]. In the case described, delayed presentation necessitated late surgical debridement. There was no need for amputation; however, the fatty degeneration caused by the grease in the muscles was notable. In addition, the grease was extensively spread throughout the tissue planes. Catheter irrigation may be useful in such cases for both infection control and removal of residual foreign material [17].

Mercury exists in nature in various forms: elemental, inorganic, and organic mercury [18]. Most severe cases of mercury toxicity are observed after inhalation of elemental mercury vapors [9]. Subcutaneous mercury can cause local necrosis, abscess, granuloma formation, and fat necrosis, but systemic toxicity is rare because systemic absorption is very low [8–10]. The greatest risk of systemic toxicity following subcutaneous or intravenous injection of elemental mercury is embolism. Pulmonary embolism and systemic toxicity have been reported in the literature [19, 20]. X‐rays are almost always useful in detecting subcutaneous elemental mercury. Laboratory mercury levels in blood and urine are valuable for diagnosis and treatment follow‐up. Surgical removal of subcutaneous mercury is recommended because of its effect on local tissues and the risk of toxicity due to slow absorption or embolization [2, 9, 10]. Because of the high toxicity of elemental mercury vapor when inhaled, it is very important for healthcare workers to use protective equipment during surgery. In addition, the risk of mercury embolism from surgical trauma should be considered. Chelation therapy is an important part of medical treatment. Dimercaprol (or British anti‐lewisite, BAL), 2,3‐DMSA, and 2,3‐dimercaptopropane‐1‐sulfonate (DMPS) are currently used as chelating agents in mercury poisoning [20, 21]. The abscess is often sterile, but broad‐spectrum antibiotics are still recommended. Patients should also be closely monitored for systemic toxicity. Pulmonary mercury embolism responds well to early diagnosis, chelation, and respiratory support therapy. Thus, such patients should be followed up in a multidisciplinary manner for chronic toxicity findings, especially in the central nervous system, gastrointestinal system, and renal system [21].

Several prognostic factors influence the outcome of hand injection injuries, including the type of injected material, injection pressure, volume of injected substance, location of injury, and delay in surgical treatment. Early surgical decompression and removal of foreign material are considered critical to prevent tissue necrosis and amputation; however, late presentation may occasionally occur. In such situations, a more controlled surgical approach aimed at infection control and restoration of circulation may be considered instead of immediate aggressive surgical intervention. In addition, management of injection injuries involving toxic substances may require a multidisciplinary approach, including plastic surgery, toxicology, infectious diseases, and rehabilitation specialists.

## Funding

The authors have nothing to report.

## Consent

Written informed consent was obtained from all patients for publication of their clinical information and images.

## Conflicts of Interest

The authors declare no conflicts of interest.

## Data Availability

The data that support the findings of this study are available from the corresponding author upon reasonable request.
